# Mitochondrial dysfunction in Parkinsonian mesenchymal stem cells impairs differentiation

**DOI:** 10.1016/j.redox.2017.10.016

**Published:** 2017-10-20

**Authors:** Plamena R. Angelova, Mario Barilani, Christopher Lovejoy, Marta Dossena, Mariele Viganò, Agostino Seresini, Daniela Piga, Sonia Gandhi, Gianni Pezzoli, Andrey Y. Abramov, Lorenza Lazzari

**Affiliations:** a Department of Molecular Neuroscience, UCL Institute of Neurology, London, UK; b Unit of Regenerative Medicine – Cell Factory, Fondazione IRCCS Ca' Granda Ospedale Maggiore Policlinico, Milan, Italy; c EPIGET LAB, Department of Clinical Sciences and Community Health, Università degli Studi di Milano, Milan, Italy; d Fondazione Grigioni per il Morbo di Parkinson, Milano, Italy; e Medical Genetics Laboratory, Fondazione IRCCS Ca' Granda Ospedale Maggiore Policlinico, Milan, Italy; f Dino Ferrari Centre, Neuroscience Section, Department of Pathophysiology and Transplantation (DEPT); g University of Milan, Neurology Unit, Fondazione IRCCS Ca' Granda Ospedale Maggiore Policlinico, Milan, Italy

**Keywords:** Parkinsonism, Progressive supranuclear palsy, Mesenchymal stem cells

## Abstract

Sporadic cases account for 90–95% of all patients with Parkinson's Disease (PD). Atypical Parkinsonism comprises approximately 20% of all patients with parkinsonism. Progressive Supranuclear Palsy (PSP) belongs to the atypical parkinsonian diseases and is histopathologically classified as a tauopathy. Here, we report that mesenchymal stem cells (MSCs) derived from the bone marrow of patients with PSP exhibit mitochondrial dysfunction in the form of decreased membrane potential and inhibited NADH-dependent respiration. Furthermore, mitochondrial dysfunction in PSP-MSCs led to a significant increase in mitochondrial ROS generation and oxidative stress, which resulted in decrease of major cellular antioxidant GSH. Additionally, higher basal rate of mitochondrial degradation and lower levels of biogenesis were found in PSP-MSCs, together leading to a reduction in mitochondrial mass. This phenotype was biologically relevant to MSC stemness properties, as it heavily impaired their differentiation into adipocytes, which mostly rely on mitochondrial metabolism for their bioenergetic demand. The defect in adipogenic differentiation was detected as a significant impairment of intracellular lipid droplet formation in PSP-MSCs. This result was corroborated at the transcriptional level by a significant reduction of PPARγ and FABP4 expression, two key genes involved in the adipogenic molecular network. Our findings in PSP-MSCs provide new insights into the etiology of ‘idiopathic’ parkinsonism, and confirm that mitochondrial dysfunction is important to the development of parkinsonism, independent of the type of the cell.

## Highlights

• PSP pathology leads to inhibition of mitochondrial respiration and decrease mitochondrial membrane potential.
• Mitochondrial dysfunction in PSP-MSCs induces ROS generation and oxidative stress.
• Higher rate of mitophagy reduces mitochondrial mass in PSP-MSCs.
• PSP impairs differentiation properties in MSCs.

## Graphical abstract

**Figure:**
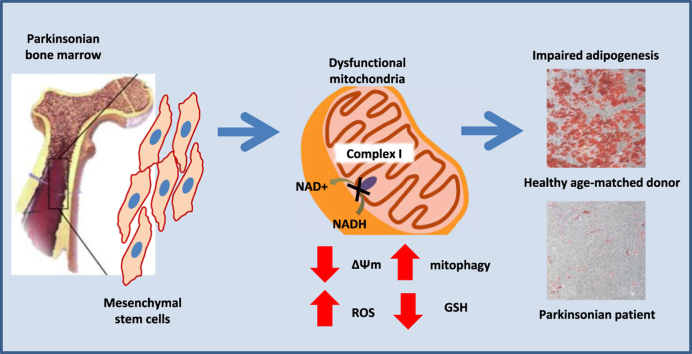

## 1 Introduction

In the framework of neurodegenerative diseases, “sporadic” or “idiopathic” forms of Parkinson's Disease (PD) and to a greater extent atypical parkinsonian disorders, have been largely neglected due to the absence of a clear and unequivocal genetic background, such as in familial monogenic PD [1]. Yet, these forms are notable for their prevalence: of 6.3 million PD patients worldwide, “idiopathic” cases account for 90–95% [2], and atypical parkinsonian disorders represent 20% of total Parkinsonism patients [3].

Atypical parkinsonism comprise a heterogeneous set of disorders sharing the symptoms found in PD, and sharing the most prominent pathological hallmark of loss of dopaminergic neurons of the substantia nigra [4]. Importantly, in contrast to PD, atypical Parkinsonism patients do not respond, or respond only transiently, to dopamine replacement therapy [4], [5], [6]. Unfortunately, as in “idiopathic” PD forms, no substantial advances have been made regarding the pathophysiology of atypical Parkinsonism. At the same time, a growing body of literature points towards metabolism as a promising avenue that may unravel novel biological pathways to neurodegeneration [7]. In particular, a major involvement of mitochondria in the pathological mechanisms of diverse neurodegenerative diseases is becoming increasingly evident [8]. This aspect has already been proven useful at the clinical level, relying on glucose tissue distribution to differentiate Parkinsonism subtypes through metabolic brain imaging [9].

In the context of atypical parkinsonian disorders, progressive supranuclear palsy (PSP), Steele–Richardson–Olszewsky syndrome (SR) type, belongs to the family of tauopathies, as it involves the accumulation of abnormally phosphorylated tau protein in both neurons and glia of cortical and subcortical structures [10]. Although a recent genome-wide association study identified genetic risk variants for the development of PSP [11], the genetic background remains necessary but not sufficient to cause this condition (since two-third of the general non-affected population share this genetic background). The most consistent genetic association involves the H1 haplotype of the tau protein-encoding MAPT gene, found in over 90% of the PSP patients [12]. Intriguingly, whereas levodopa response is poor or absent, PSP patients have shown slight improvements after treatment with coenzyme Q10, a key component of the mitochondrial electron transport chain (ETC) and a crucial radical-scavenging antioxidant [13]. Very recently, within a phase I clinical study (trial registration NCT01824121 at https://clinicaltrials.gov/) we evaluated the safety of autologous bone marrow mesenchymal stem cell (MSC) administration to PSP patients to stabilize the progression of the disease, thanks to MSC paracrine properties [14]. This posed a unique opportunity to address whether PSP patient-derived MSCs could recapitulate general non-neural cell aspects of Parkinsonism, such as mitochondrial dysfunction. For the first time in the literature, our study will provide novel insights on disease mechanisms in the stem cell compartment of these patients and will investigate its consequences on their differentiation properties.

Based on this premise, a comprehensive study focused on mitochondrial function was performed to unveil novel metabolism-related clues that may shed light on PSP pathophysiology. Importantly, to avoid effects associated with the most acknowledged genetic susceptibility factors, and to obtain data on new pathological mechanisms, we used MSCs derived only from PSP patients screened against the MAPT H1 haplotype. With this approach, we aimed at obtaining insights on “idiopathic” Parkinsonism etiology, which would contribute to a comprehensive understanding of parkinsonian disorders as a whole.

## 2 Materials and methods

### 2.1 Cell culture

MSCs were isolated from bone marrow (BM) aspirates seeding 50,000 mononucleated cells/cm^2^ in αMEM (Thermo Fisher Scientific, Waltham, MA, USA) supplemented with 10% fetal bovine serum (FBS; Thermo Fisher Scientific), in T75 flasks. The cultures were incubated at 37 °C, 20% O_2_, 5% CO_2_. Medium changes were performed twice a week. Two weeks after initial seeding, primary BMMSC colonies were detached with a 10 min incubation at 37 °C with TrypLE Select Enzyme (Thermo Fisher Scientific) and replated at 4000 cells/cm^2^ in the same medium. BMMSC identity was previously assessed [29]. Subsequent passages were performed following the same steps. Passage 4–6 BMMSCs were used for all experiments. BM from Progressive Supranuclear Palsy (PSP) patients was collected in the context of a clinical protocol authorized by the local Ethics Committee of Fondazione IRCCS Ca’ Granda Ospedale Maggiore Policlinico (Italy), by the national competent authority for phase-I cell therapy at the National Health Institute (Istituto Superiore di Sanità) and approved by the Italian Medicines Agency (Agenzia Italiana del Farmaco, AIFA). The trial is registered at ClinicalTrials.gov (NCT01824121). BM from healthy donors was collected in the context of the EU-funded REBORNE project. All BM donors gave their written informed consent.

### 2.2 Live cell imaging

For identification of mitochondrial and lysosomal localization, cells were loaded with 200 nM MitoTracker Green FM and 50 nM LysoTracker Red in HBSS for 30 min before experiments. Confocal images were obtained using a Zeiss 710 confocal microscope equipped with 40X oil immersion objective. The 488 nm Argon laser line was used to excite MitoTracker Green fluorescence which was measured between 505 and 530 nm. Illumination intensity was kept to a minimum (about 1% of laser output) to avoid phototoxicity and the pinhole set to give an optical slice of ~2 µm. For LysoTracker Red, the 543 nm Ne/He laser line was used with measurement above 650 nm. All data presented were obtained from at least 5 coverslips and 2–3 different cell preparations.

For measurements of ΔΨ_m_, cells plated on 22 mm glass coverslips were loaded for 30 min at room temperature with 25 nM tetramethylrhodamine methylester (TMRM; Invitrogen) in a HEPES buffered saline solution (HBSS) composed of 156 mM NaCl, 3 mM KCl, 2 mM MgSO_4_, 1.25 mM KH_2_PO_4_, 2 mM CaCl_2_, 10 mM glucose and 10 mM HEPES; pH adjusted to 7.35 with NaOH. The dye remained present in the media at the time of recording. Confocal images were obtained using a Zeiss 710 VIS CLSM equipped with a META detection system and a 40x oil immersion objective. TMRM was excited using the 560 nm laser line and fluorescence was measured above 580 nm. For basal ΔΨ_m_ measurements, Z-stack images were obtained by confocal microscopy and analysed using Zeiss software. For analysis of response to mitochondrial toxins, images were recorded continuously from a single focal plane. TMRM is used in the redistribution mode to assess ΔΨ_m_, and therefore a reduction in TMRM fluorescence represents mitochondrial depolarisation. For measurement of mitochondrial ROS production, cells were pre-incubated with MitoTracker Red CM-H(2)XROS for 10 min at room temperature. MitoTracker Red CM-H(2)XROS measurements were produced using 560 nm excitation and emission above 580 nm.

### 2.3 Measurement of NADH/FAD redox index

NADH autofluorescence was measured using an epifluorescence inverted microscope equipped with a 20X fluorite objective. Excitation light at a wavelength of 350 nm was provided by a Xenon arc lamp, the beam passing through a monochromator (Cairn Research, Kent, UK). Emitted fluorescence light was reflected through a 455 nm long-pass filter to a cooled CCD camera (Retiga, QImaging, Canada) and digitized to 12 bit resolution. Imaging data were collected and analysed using software from Andor (Belfast, UK). FAD autofluorescence was monitored using a Zeiss 710 VIS CLSM equipped with a META detection system and a 40x oil immersion objective. Excitation was using the 454 nm Argon laser line and fluorescence was measured from 505 to 550 nm. Illumination intensity was kept to a minimum (at 0.1–0.2% of laser output) to avoid phototoxicity and the pinhole set to give an optical slice of ~2 µm. FAD and NADH redox indexes and mitochondrial pools were estimated according the method described in Bartolome et al. [46].

### 2.4 Glutathione assessments

Neuronal cultures were incubated with 50 μM monochlorobimane (MCB) (Molecular Probes, Invitrogen) for 40 min in HEPES buffered salt solution prior to imaging [47]. Cells were then washed with HEPES buffered salt solution and images of the fluorescence of the MCB-GSH were acquired using a Zeiss 710 CLSM with excitation at 405 nm and emission at 435–485 nm.

### 2.5 RNA extraction

Isolation of total RNA was obtained by TRIzol extraction. Briefly, each sample was lysed directly on the culture dish with 1 mL of TRIzol reagent (Thermo Fisher Scientific). Then, 0.2 mL of chloroform was added and the samples were mixed by inversion, incubated for 5 min and centrifuged at 11,000 rpm for 15 min at 4 °C. The resulting RNA-containing aqueous phase was collected and transferred to a new tube. Next, 0.5 mL of isopropanol was added. After 10 min incubation, the samples were centrifuged at 11,000 rpm for 10 min at 4 °C. The supernatant was discarded and the precipitated RNA resuspended in 1 mL of 75% ethanol, mixed by vortexing and centrifuged at 9000 rpm for 5 min at 4 °C. Finally, the supernatant was discarded and the purified precipitated RNA air dried for 10 min and resuspended in 20 μL of RNase-free water. RNA concentration and quality were determined by reading the absorbance at 260 nm with a Nanodrop 1000 spectrophotometer (Thermo Fisher Scientific) and checking A260/A230 and A260/A280 ratios. RNA integrity was addressed by agarose gel electrophoresis.

### 2.6 Real Time qRT-PCR

800 ng of RNA were retrotranscribed using the iScript cDNA synthesis kit (Bio-Rad, Hercules, CA, USA), following manufacturer's instructions. Real Time qRT-PCR assays were conducted in triplicate using SsoFast EvaGreen Supermix (Bio-Rad), following manufacturer's instructions and preparing 10 μL amplification reactions. The thermal profile used was: 98 °C for 5 min, 46 cycles of 95 °C for 5 s and 60 °C for 20 s. Specificity of amplification was checked by assessing single peak amplification curves. Transcript levels were normalized on TBP and GAPDH “house-keeping” genes, following the 2^ΔΔCt^ method, using healthy MSCs as control. Primer sequences for all assays will be given upon request.

### 2.7 DNA extraction

Isolation of total DNA was performed by GentraPuregene Blood Kit (Qiagen, Hilden, Germany), following manufacturer's instructions. Briefly, cells were lyzed with 300 μL cell lysis solution and vortexed. Proteins were precipitated with 100 μL protein precipitation solution and pelleted at 13,000 × *g* for 1 min. The supernatant was collected, mixed with 300 μL isopropanol and centrifuged at 13,000 × *g* for 1 min. The supernatant was discarded and the DNA pellet was washed with 70% ethanol and centrifuged at 13,000 × *g* for 1 min. The resulting DNA pellet was resuspended in 50 μL hydration solution. DNA concentration and quality were assessed by reading the absorbance at 260 nm with a Nanodrop 100 spectrophotometer (Thermo Fisher Scientific) and checking A260/A230 and A260/A280 ratios. DNA integrity was addressed by agarose gel electrophoresis.

### 2.8 Nuclear DNA sequencing analysis

To analyze genomic DNA extracted from both PSP- and control MSCs, a targeted resequencing approach using Next-Generation Sequencing (NGS) technique was implemented. Target enrichment for all the samples was performed using HaloPlex Target Enrichment System kit for Illumina Sequencing, 48 rxn (Agilent Technologies, Santa Clara, CA, USA), according to the manufacturer's protocol. A total of 225 ng genomic DNA per sample was digested in eight different restriction reactions (30 min incubation at 37 °C). The eight digestion reactions were combined into a single hybridization mix containing target specific probes and a specific HaloPlex index (8 nt oligonucleotides) for each sample. Each index univocally identifies each sample during the sequencing. Hybridization reactions were performed in 3-h incubations at 54 °C. Probes were labeled with biotin and designed to hybridize to both ends of the digested fragments, therefore generating circular fragments containing one nick. Then, the DNA probe hybrids were captured with streptavidin beads to eliminate linear, non-target DNA fragments. With a ligation reaction, the remaining nicks were closed to complete circularization. The captured DNA was eluted from the magnetic beads with 50 mM NaOH and amplified by PCR reaction, followed by a final purification reaction with AMPure XP magnetic beads (Beckman Coulter, Brea, CA, USA). Finally, the concentration of each library was measured using 2200 TapeStation instrument (Agilent Technologies, Santa Clara, CA, USA) and, after the appropriate dilution of each sample with TE buffer, final 4 nM libraries were obtained. Then the libraries were pooled and after further dilutions a 7 pM final pool was obtained (this is the ideal concentration to achieve a cluster density of about 900/1000 K/mm^2^ on the flow cell). The final purified, quantified and pooled multiplex PCR products were sequenced on MiSeq platform (Illumina, San Diego, CA, USA) with a 2 × 150 bp paired-end sequencing, using MiSeq reagent kit V2. Target genes analysed were: SNCA, PARK2, UCHL1, PINK1, DJ1, LRRK2, GBA, VPS35, ATP13A2, EIF4G1, HTRA2, DNAJC13, VPS13C, DNAJC6, FBXO7, PLA2G6, SYNJ1 and MAPT.

### 2.9 Mitochondrial DNA sequencing analysis

Molecular analysis was performed on the genomic DNA extracted from control and PSP-MSCs. According to a standardized protocol, the entire mitochondrial (mt) DNA was amplified by PCR into 46 overlapping fragments using a set of coupled primers (Variant SEQr™ Resequencing System Applied Biosystems). PCR products were purified and each fragment was then sequenced, with two specific primers M13 forward and reverse, and analysed using an ABI PRISM 3130xl Genetic Analyzer.

### 2.10 Evaluation of MSC adipogenic potential

In order to promote differentiation, a defined adipogenic medium (Lonza, Basel, Switzerland) was used on MSCs from healthy controls and PSP patients at passages P3. MSCs were seeded at a density of 3 × 10^4^ cells/cm^2^ in αMEM-GlutaMAX (Thermo Fisher Scientific) with 10% fetal bovine serum (Thermo Fisher Scientific). The day after, three cycles of differentiation induction and maintenance followed. Briefly, the cycle consisted in 3 days in human MSC Adipogenesis Induction medium (Lonza), followed by 3 days in human MSC Adipogenesis Maintenance medium (Lonza). A final maintenance cycle of 7 days concluded the differentiation protocol, during which medium was changed every 2–3 days. Then, adipogenic differentiation was detected by microscopic observation, and lipid vacuoles were stained with fresh Oil Red O solution (Sigma-Aldrich, Saint Louis, MI, USA). Briefly, differentiated MSCs were washed with PBS (Thermo Fisher Scientific) and then fixed with paraformaldehyde (4% in water) for 1 h at RT. After a washing step with PBS, differentiated MSCs were stained with fresh Oil Red O working solution, prepared diluting 3 parts of a 0.5% isopropanol stock solution with additional 2 parts of water. Three final washing steps with PBS followed. Finally, Oil Red O incorporation was quantified biochemically. To recover Oil Red O, dry wells containing differentiated MSCs were incubated with isopropanol for 1 h. To determine the amount of extracted Oil Red O, the absorbance of the samples were read at 492 nm using a GENios plus plate reader (Tecan, Männedorf, Switzerland).

### 2.11 Data and statistical analysis

Data analysis was performed using Origin 8 (Microcal Software Inc., Northampton, MA) software. Statistical tests: unpaired two-tailed Student's tests were performed using GraphPad Prism software (GraphPad Software, Inc. La Jolla, USA). Differences were considered statistically significant with p-value < 0.01. Results are expressed as means ± standard error of the mean (S.E.M.).

## 3 Results

### 3.1 PSP-MSCs exhibit decreased mitochondrial membrane potential

**Fig. 1:**
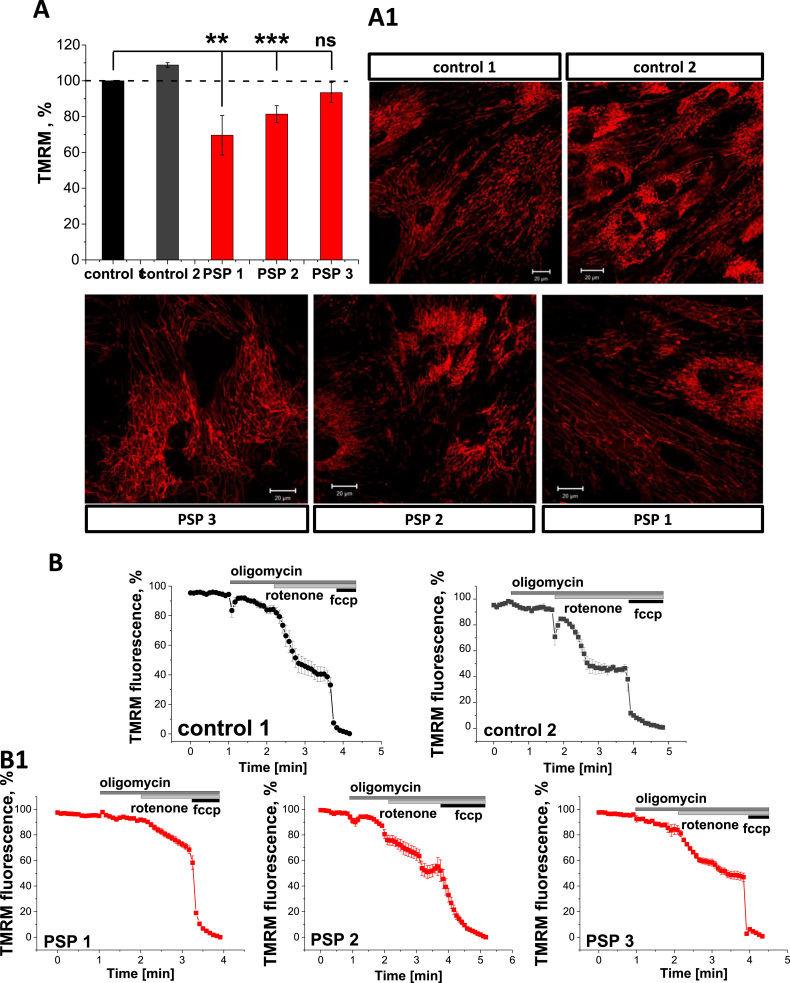
Mitochondrial membrane potential is affected in PSP-MSCs. A. Histogram comparing the ΔΨ_m_ values of control (black, dark grey) and patients (red) MSC. **A1.** Images show mitochondrial networks in control and patient MSCs. ΔΨ_m_ was assessed with TMRM (red). Assessment of the mitochondrial respiratory chain function and the maintenance of ΔΨ_m_ in control (B) and patient (B1) MSCs. (For interpretation of the references to color in this figure legend, the reader is referred to the web version of this article.)

Mitochondrial membrane potential (ΔΨ_m_) is an important indicator of mitochondrial health. Using TMRM as a fluorescent indicator of ΔΨ_m_, we found that PSP-MSCs showed significantly decreased basal ΔΨ_m_ (Fig. 1, A and A1). Thus, in PSP-MSC 1, ∆Ψ_m_ was reduced to 69.6 ± 11.0% (n = 20; p < 0.001) of the control age-matched MSCs, and PSP-MSC 2 and PSP-MSC 3–81.4 ± 4.7% (n = 20) and 79.9 ± 9.3% (n = 20) of control, respectively. Therefore, in the context of PSP, mitochondrial function in stem cells is likely to be impaired.

In the majority of healthy cells, ΔΨ_m_ is maintained by the activity of the mitochondrial respiratory chain. In the event of damage or inhibition of respiration, cells may maintain mitochondrial membrane potential using ATP hydrolysis by the ATP synthase. In order to investigate the mechanism of maintenance of ΔΨ_m_, we applied a series of mitochondrial toxins and observed their effects on ΔΨ_m_. In both control MSCs, application of oligomycin (2 µg/mL), an inhibitor of the F_1_F_0_-ATPase, induced no response or a slight depolarisation, as proton entry through the ATP synthase was inhibited. Application of rotenone (5 µM) to inhibit complex I then produced a profound depolarisation, and complete depolarisation was estimated by addition of the mitochondrial uncoupler FCCP (1 µM; Fig. 1, B). This indicates that in control MSCs most of the mitochondrial membrane potential is maintained by the respiratory chain. In PSP-MSCs, application of oligomycin also induced only minor depolarisation (3–5%) or no effect (Fig. 1, B1), indicating that the ΔΨ_m_ is still maintained by the mitochondrial respiratory chain and suggesting that, even under conditions of low ΔΨ_m_, the ATP synthase in PSP-MSCs is unable to switch to hydrolysis mode. Importantly, the effect of rotenone on ΔΨ_m_ of PSP-MSCs was slower and smaller compared to control MSCs (from ~60% in control MSCs to only ~40% in PSP-MSCs), suggesting partial inhibition of the complex I in the patient MSCs (Fig. 1, B1). Complete depolarisation was achieved also in PSP-MSCs after FCCP addition (Fig. 1, B1).

### 3.2 PSP-MSCs demonstrate inhibition of NADH-dependent respiration

**Fig. 2:**
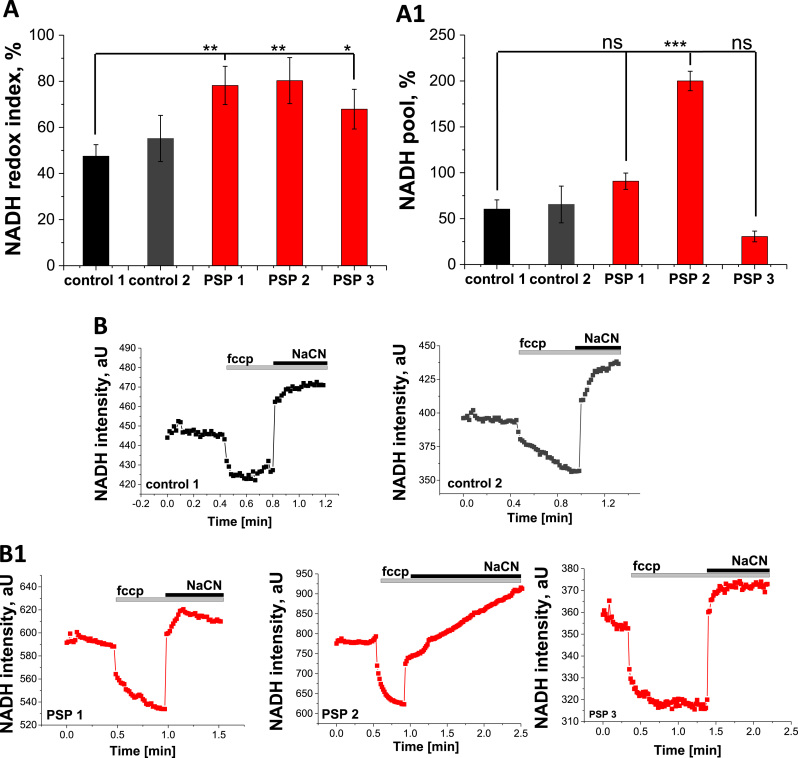
PSP leads to inhibition of NADH-dependent respiration. A. Quantification of the NADH redox index from control (black, dark grey) and patient MSCs (red). **A1**. NADH pool**,** calculated from control (black, dark grey) and patient MSCs (red). Representative traces from control **(black traces, B)** or patient MSCs (red traces, **B1**) NADH autofluorescence. Application of 1 µM fccp maximizes respiration and addition of 1 mM NaCN fully inhibits mitochondrial respiration. (For interpretation of the references to color in this figure legend, the reader is referred to the web version of this article.)

In order to investigate mitochondrial respiration specifically in the cells with lower ΔΨ_m_ and smaller response to rotenone, NADH autofluorescence was measured in PSP- and control MSCs and the redox index and relative size of the pool of NADH in mitochondria were calculated. NADH is the electron donor for complex I, and as such, NADH levels correlate inversely with respiratory chain activity. In order to measure redox index, we applied FCCP (1 µM) to maximise respiration and therefore minimise the NADH pool, then added NaCN (1 mM) to block mitochondrial respiration and consequently maximise the NADH pool. The initial NADH autofluorescence is then calculated as a percentage of this range (Fig. 2, A). In addition, the total mitochondrial pool of NADH may be taken as an indication of the substrate availability for complex I (Fig. 2, A1).

We observed a significant increase in redox levels in PSP-MSCs (from 47.5 ± 5.0%, n = 17, for control MSC 1 and 55.2 ± 10.0%, n = 19, for control MSC 2, to 78.2 ± 8.3%, n = 10, p = 0.0024, for PSP-MSC 1, 83.5 ± 10.0%, n = 9, p = 0.0031, for PSP-MSC 2 and 67.9 ± 8.6%, n = 8, p = 0.0396, for PSP-MSC 3; Fig. 2, A), indicating decreased respiration rate in these cells. The total mitochondrial pool of NADH in PSP-MSCs was also higher than in control MSCs (from 60.4 ± 10.0%, n = 17, for control MSC 1 and 65.5 ± 20.0%, n = 19, for control MSC 2, to 90.8 ± 8.9%, n = 10, p=0.0501, for PSP-MSC 1 and 200.0 ± 10.6%, n = 9, p < 0.0001, for PSP-MSC 2; Fig. 2, A1), indicating increased substrate availability for complex I of the ETC in these cells.

### 3.3 Mitochondrial dysfunction in PSP-MSCs induces significant increase in ROS generation and oxidative stress

**Fig. 3:**
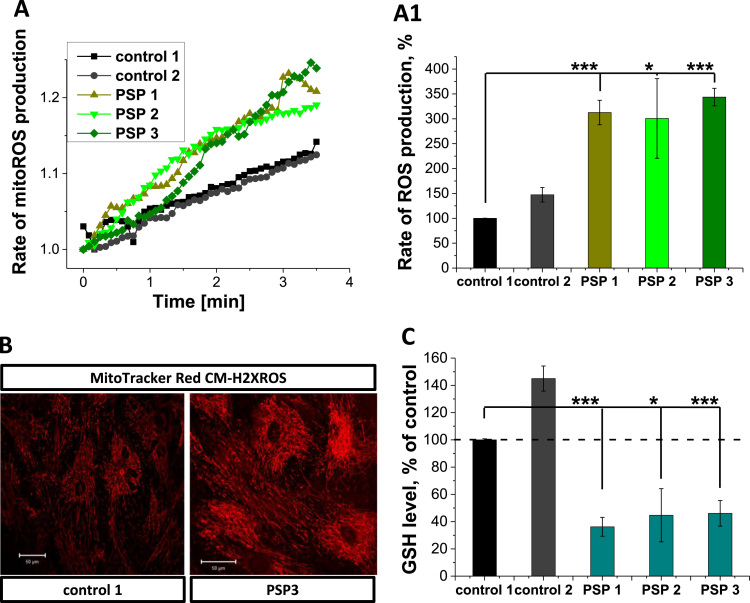
Increased ROS production and decreased GSH levels in PSP-MSCs. Representative traces for the rate of mitochondrial reactive oxygen species (ROS) generation (**A.**) and quantification of these experiments (**A1**.) in patient (light green, green, olive) and control (black, dark grey) MSCs. **B.** Representative images of control and patient MSCs, labeled with MitoTracker Red CM-H2XROS. **B1.** Levels of Glutathione (GSH), assessed by MCB fluorescence- controls (black, dark grey) and patient MSCs (teal). (For interpretation of the references to color in this figure legend, the reader is referred to the web version of this article.)

Mitochondrial ROS production is linked to the rate of respiration and therefore also to the mitochondrial membrane potential [15]. Moreover, inhibition of the complex I contributes to higher rates of ROS production [16]. The basal rates of mitochondrial ROS production, as measured with the mitochondria-specific probe MitoTracker Red CM-H2xROS, differed between the control MSCs and PSP-MSCs (Fig. 3, A, A1 and B). Thus, the rates of ROS production in PSP-MSC 1, PSP-MSC 2 and PSP-MSC 3 were 3-fold higher (312.6 ± 24.6%, n = 9, p < 0.0001, for PSP-MSC 1, 300.7 ± 80.1%, n = 11, p = 0.0128, for PSP-MSC 2 and 343.7 ± 17.7%, n = 8, p < 0.0001, for PSP-MSC 3) compared to control MSC 1 (100.0 ± 0.2%, n = 13; Fig. 3, A1). Such a high level of ROS production can induce oxidative stress, which results in the reduction of the level of major antioxidants. We used monochlorobimane (MCB) to measure the level of glutathione (GSH). Mitochondrial ROS overproduction in PSP-MSCs significantly decreased the level of GSH in these cells (36.2 ± 6.9%, n = 25, p < 0.0001, for PSP-MSC 1, 44.7 ± 19.5%, n = 25, p = 0.0167, for PSP-MSC 2 and 46.1 ± 9.4%, n = 25, p < 0.0001, for PSP-MSC 3) compared to control MSCs (100.0 ± 0.1%, n = 25, for control MSC 1 and 145.5 ± 9.2%, n = 25, for control MSC 2; Fig. 3, C). Thus, mitochondrial dysfunction in PSP-MSCs leads to oxidative stress.

### 3.4 Basal rate of mitophagy is higher in PSP-MSCs

**Fig. 4:**
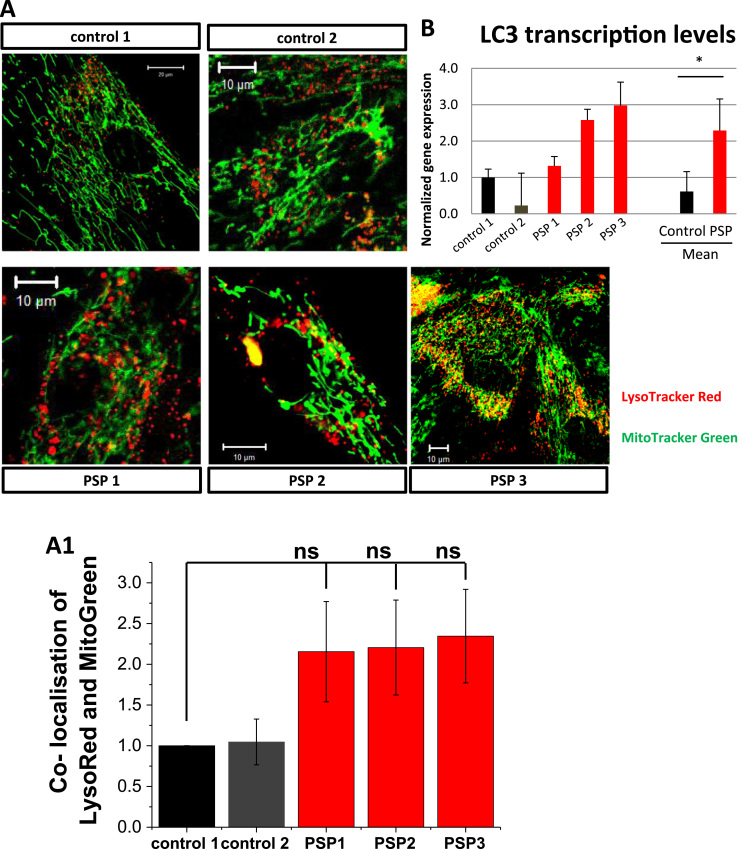
Basal mitophagy rate is increased in PSP-MSCs. A. Representative images of lysosomes labeled with LysoTracker Red (red) and mitochondria with MitoTracker Green (green). Merging of both signals is counted as a mitophagy event. **A1**. Histogram showing co-localization of LysoTracker Red with MitoTracker Green in control (black, dark grey) and patient (red) MSCs, as a sign of early mitophagy. **B.** Transcription levels of LC3 in control and PSP patient's MSCs. (For interpretation of the references to color in this figure legend, the reader is referred to the web version of this article.)

Cell death is triggered by the release of intramitochondrial proteins, therefore it is widely accepted that mitochondria are regulators of cell faith. Considering this, the removal of dysfunctional mitochondria is an important process for ensuring cell survival. Damaged mitochondria, as well as other impaired organelles and proteins, are degraded by specific mitochondrial autophagy (mitophagy). Deregulation of mitophagy can lead to an increase in the number of damaged mitochondria that can induce mitochondrial dysfunction and trigger the activation of cell death pathways. First, we addressed autophagy protein LC3 gene expression, which was four-fold higher in PSP- compared to control MSCs (p < 0.05; Fig. 4, A). Next, to measure the level of mitophagy in PSP pathology, we estimated the co-localization of the mitochondria (labeled with MitoTracker green) with the lysosomes (labeled by LysoTracker red), which allows us to measure autophagic degradation of mitochondria independently of any specific pathways [17]. We have found that percentage of co-localized mitochondria and lysosomes is much higher in PSP-MSCs (2.155 ± 0.690%, n = 25, p = 0.1563, for PSP-MSC 1, 2.205 ± 0.583%, n = 25, p = 0.1195, for PSP-MSC 2 and 2.345 ± 0.573%, n = 30, p = 0.1062, for PSP-MSC 3), compared to control MSCs (1.000 ± 0.100%, n = 15, for control MSC 1 and 1.046 ± 0.280%, n = 20, for control MSC 2; Fig. 4, B). Thus, mitochondrial dysfunction in PSP-MSCs induces higher basal rate of mitophagy.

### 3.5 Higher rate of mitophagy reduces mitochondrial mass in PSP-MSCs

**Fig. 5:**
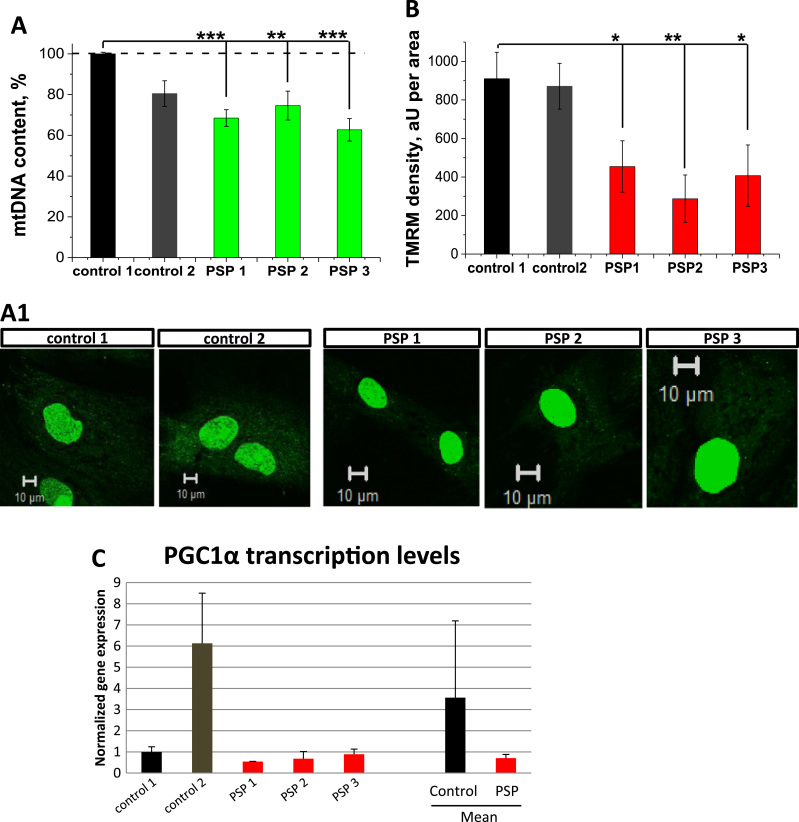
Mitochondrial biogenesis is decreased or insufficient in PSP-MSCs. A. Mitochondrial DNA content was decreased as measured by PicoGreen fluorescence. **A1** Representative images of the mtDNA content in control and patient MSCs. **B.** Mitochondrial mass is significantly reduced in patient MSCs. **C.** transcription levels of the PGC1α in control and PSP patient's MSCs.

The number of mitochondria within the cells is regulated by the balance between mitochondrial biogenesis and mitophagy. Considering this, for the normal maintenance of the mitochondrial balance, PSP-MSCs should activate mitochondrial biogenesis. However, the level of mitochondrial DNA in the cells, measured by PicoGreen fluorescence in non-nuclear area as an estimation of the number of mitochondria, was significantly lower in PSP-MSC 1 (68.5 ± 4.1%, n = 35, p < 0.0001), PSP-MSC 2 (74.6 ± 7.1%, n = 25, p = 0.0026) and PSP-MSC 3 (62.7 ± 5.5%, n = 15, p < 0.0001) compared to control MSC 1 (100.0 ± 0.2%, n = 20) and 2 (80.5± 6.3%, n = 30); (Fig. 5, A). Total mitochondrial mass (estimated as the total TMRM density in aU per area of the cell) was as well significantly lower in PSP-MSC 1 (454.2 ± 133.5, n = 30, p = 0.0215), PSP-MSC 2 (287.1 ± 123.4, n = 25, p = 0.0014) and PSP-MSC 3 (407.1 ± 159.3, n = 25, p = 0.0206), compared to control MSC 1 (910.3 ± 137.0, n = 25) and control MSC 2 (871.1 ± 119.0, n = 25), as shown in Fig. 5, B). In accordance with these results, the gene expression level of mitochondrial biogenesis master regulator *PGC1α* was five-fold higher in control compared to PSP- MSCs (Fig. 5, C).

### 3.6 PSP phenotype arises independently of known parkinsonism-related mutations

To address genetic mutations already known to participate in the development of PD or other parkinsonian pathologies, both nuclear and mtDNA were analysed. Next-generation sequencing revealed no mutations in a selected group of 18 genes known in the literature to be involved in the pathogenesis of the genetic forms of PD and other parkinsonian diseases. Synonymous or nonsynonymous single nucleotide variants known to be single nucleotide polymorphisms or benign variants were observed. Minor variants with uncertain clinical relevance were found in both PSP- and control MSCs. Similarly, sequencing analysis of the entire mtDNA did not reveal pathogenic mutations among all PSP- and control MSCs. Several homoplasmic variants were detected, which, again, are known to be benign polymorphisms. Thus, no known genetic mutation determines the observed PSP phenotype.

### 3.7 PSP-MSCs show impaired differentiation properties compared to age-matched healthy controls

**Fig. 6:**
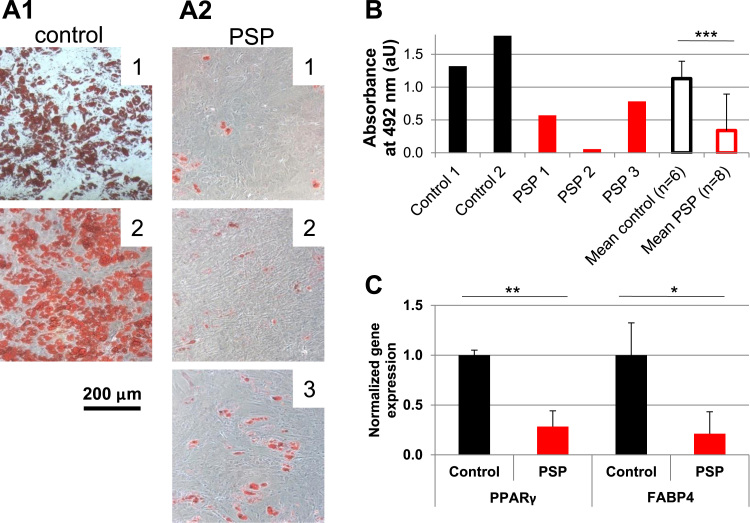
Differentiation potential is impaired in PSP-MSCs. A1. Oil Red O-positive adipocytes in MSCs from healthy donors. **A2.** Lack of abundant adipogenic differentiation in PSP-MSCs, as evidenced by fewer Oil Red O-positive cells. **B.** Biochemical quantification of incorporated Oil Red O in control and PSP-MSCs. Absorbance values measured at 492 nm expressed as arbitrary units (aU). **C.** PSP-MSCs express significantly lower levels of adipogenesis-related PPARγ and FABP4 transcripts compared to age-matched controls; gene expression in normalized to control MSCs. Control, healthy MSCs; PSP, PSP-MSCs.

Based on the hypothesis that mitochondrial activity is pivotal for MSC differentiation capability, adipogenic potential was assessed, being the most energy-demanding differentiation process that mainly relies on mitochondrial activity. After a 3 weeks differentiation protocol on a large group of PSP-MSCs (n = 8) and age-matched healthy control MSCs (n = 6), adipogenic differentiation was evaluated by Oil Red O staining and by biochemical quantification of its incorporation into differentiated cells. As shown in Fig. 6A1, control MSCs differentiated steadily and homogeneously into mature adipocytes, containing Oil Red O-positive large fat droplets. On the other hand, PSP-MSCs failed to reach full adipogenic differentiation, presenting fewer Oil Red O-positive adipocytes and smaller fat deposits. Biochemical quantification of incorporated Oil Red O in the different samples confirmed more accurately this result, demonstrating that PSP-MSCs had significantly reduced adipogenic potential compared to control MSCs (p < 0.005). Moreover, this aspect was confirmed at the gene expression level. Adipogenesis regulators PPARγ and FABP4 were significantly downregulated in PSP- compared to control MSCs (p < 0.005 and p < 0.05, respectively; Fig. 6C).

## 4 Discussion

Stem cell metabolism is a growing area of research in the stem cell field. The influence of mechanisms of energy production on stem cell physiology and disease is increasingly recognized. Specifically, metabolism has been associated with stem cell self-renewal and differentiation control. For instance, high glucose was shown to suppress embryonic stem cell differentiation into neural cells [18]; hypoxia was shown to sustain stemness by inhibition of oxidative phosphorylation and increased glycolysis by activation of HIF-1α and consequent inhibition of pyruvate dehydrogenase kinase activity [19]. Intriguingly, it was also observed that, during stem cell asymmetric divisions, old mitochondria segregate with daughter cells fated to differentiation, whereas during symmetric divisions new and old mitochondria are equally distributed [20]. This way, mitochondria themselves appear to play a central role in stem cell fate decisions. Concerning the influence of mitochondria on MSC differentiation potential, it was demonstrated that mitochondrial respiration influences adipogenic properties of MSCs. Indeed, increased mitochondrial activity is a prerequisite for the maturation of adipocytes from MSCs, since the knockdown of the mitochondrial transcription factors resulted in the inhibition of adipogenesis in MSCs [21]. Furthermore, a study recently showed that adipogenesis differentiation involves MSC mitochondrial fusion and consequent increased expression of fusion protein Mfn 1 and 2 [22].

In this context, the metabolism of MSCs of neurodegenerative disease patients has still not been addressed, until now. Understanding the basis of the crosstalk between mitochondria and cell fate is of critical importance, given the promising application of stem cells in regenerative medicine. Furthermore, MSCs are widely recognized as regulators of tissue homeostasis [23], and it is therefore of utmost importance to determine the influence of neurodegenerative-associated metabolism on stem cell function. Some reports have shown widespread systemic involvement for specific neurodegenerative diseases, where non-neural cells are affected or influenced the pathological neuronal loss. For instance, alterations in metalloproteases, crucial actors in tissue remodeling and stem cell mobilization, were found in the MSC compartment in amyotrophic lateral sclerosis (ALS) patients [24]. A further two studies demonstrated a correlation between ALS progression rate and impaired MSC function [25], [26], reporting that functional deficiencies in MSCs were proportional to disease progression. Moreover, MSC impaired functionality was also observed in multiple sclerosis (MS) patients [27]. In detail, conditioned media from MSCs of patients with long duration of progressive disease showed reduced neuroglial protective capacity. This observation led to the identification by mass spectrometry of 40 factors whose secretion was altered in progressive MS. Finally, hTERT-immortalized adipose tissue-derived MSCs from idiopathic or familial PD patients showed decreased activity in mitochondrial complexes I, II and IV [28], suggesting that such cells could represent a good model to study mitochondrial dysfunctions of PD pathogenesis and treatment.

Thus, this study represents the first in its effort to describe the effects of a parkinsonian disease in the bone marrow stem cell compartment, from the perspective of MSC bioenergetics. The rationale of the study was to evaluate if metabolic dysfunction observed in the mitochondria in patient neurons were also present in a distant stem cell compartment. This study is of paramount importance, due to the currently proposed use of autologous MSCs in the treatment of neurodegenerative diseases [29]. Indeed, our data indicate that MSCs from the bone marrow of PSP patients show impaired mitochondrial functionality, still in the context of useful neuroprotective factor secretion. Therefore, their use in preliminary clinical studies should be thoroughly evaluated, taking into account also their metabolic state, which could be correlated to clinical outcome effectiveness.

Specifically, we observed a decrease in the mitochondrial membrane potential of PSP patient MSCs (PSP-MSCs) and an increase of NADH redox state, suggesting a partial inhibition of complex I, as already described by some reports for PSP patients [30]. Importantly, this hypothesis was confirmed in further experiments, where the effect of complex I inhibitor rotenone on mitochondrial membrane potential was significantly lower in PSP-MSCs. Mitochondrial membrane potential in these conditions was still predominantly maintained by electron transport chain (by activation of the complex II, a mechanism reported by the literature [31], [32], [33], [34], [35] and also by others) due to almost similar responses to ATP synthase inhibitor oligomycin and much larger responses to an uncoupler agent. In all the experiments the comparison to healthy age-matched bone marrow donors allowed us to exclude the possibility that the observed results were related to an age-specific phenotype.

Activation of the complex II in the context of partial inhibition of mitochondrial complex I can lead to increased production of ROS in mitochondria [16], [36]. Although mitochondrial ROS can play a physiological role [37], extremely high ROS production in mitochondria of PSP-MSCs induced high levels of oxidative stress, which resulted in a significant decrease of the major endogenous antioxidant GSH.

Oxidative damage can trigger mitophagy, a form of mitochondria-specific autophagy [38], [39], [40], [41], [42], [43]. In PSP-MSCs, higher co-localization of the mitochondria and lysosomes was observed suggesting a higher rate of mitophagy compared to age-matched healthy controls. This difference could be due to the aforementioned mitochondria-driven oxidative stress. This higher rate of mitophagy was coupled with a decreased or insufficient rate of mitochondrial biogenesis, which led to an overall lower mitochondrial content in PSP-MSCs.

Given the crucial role played by healthy mitochondria on adipogenic differentiation, as earlier described, we tested if impaired mitochondrial activity could have detrimental effects on PSP-MSC properties. Strikingly, all PSP-MSCs showed a reduced capacity to generate fully differentiated adipocytes, confirming for the first time the impairment of the stem cell compartment differentiation properties in a parkinsonian disease.

It is important to note that a lower mitochondrial density in combination with inhibition of mitochondrial complex I is not enough to induce severe pathology of energy metabolism under resting conditions. Yet it may be limiting and pathological under the increased energy demand of differentiation to specific cells, such as adipocytes or functional neurons.

It is reported in the literature that PSP pathology is related to mitochondrial complex I dysfunction. In the absence of a known mutation, as in our samples screened against the main MAPT gene PSP-associated H1 haplotype, it is possible that minor mutations at the mtDNA level could negatively influence complex I activity, as proposed by some reports [30], [44], [45]. In these studies, transmitochondrial cytoplasmic hybrids (cybrids) cell lines, expressing PSP mtDNA, recapitulated a bioenergetic defect similar to that of PSP cells: higher oxidative stress, higher anti-oxidant activity, impaired complex I activity. Notwithstanding, we cannot exclude that the nature of such defects could be that of a “secondary” damage related to systemic consequences of the neurodegenerative disease localized at the brain tissue, rather than a “primary” damage, directly associated to PSP aetiopathology. Yet, no relevant genomic alterations were found in nuclear or mitochondrial DNA of our samples, which could be attributed to the development of the parkinsonian disease phenotype.

In conclusion, despite the traditional view of Parkinsonism as central nervous system diseases, here, for the first time in the literature, we unexpectedly detected severe mitochondrial function impairment in stem cells isolated from the bone marrow of PSP patients. Therefore, our conclusion is that crucial metabolic aspects of parkinsonian disorders are indeed recapitulated at the systemic level, in compartments other than the nervous system.

## Author contributions

Author contribution: P.R.A.: conception and design, collection and assembly of data, data analysis and interpretation, manuscript writing, final approval of manuscript; M.B.: conception and design, collection and assembly of data, data analysis and interpretation, manuscript writing, final approval of manuscript; C.L.: collection and assembly of data, final approval of manuscript; M.D.: collection and assembly of data, final approval of manuscript; M.V.: collection and assembly of data, final approval of manuscript; A.S.: collection and assembly of data, final approval of manuscript; D.P.: collection and assembly of data, final approval of manuscript; G.P.: final approval of manuscript; S.G: conception and design, final approval of manuscript. A.Y.A.: conception and design, manuscript writing; final approval of manuscript; L.L.: conception and design, final approval of manuscript.
